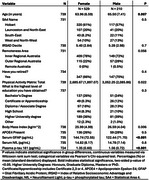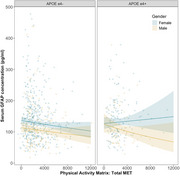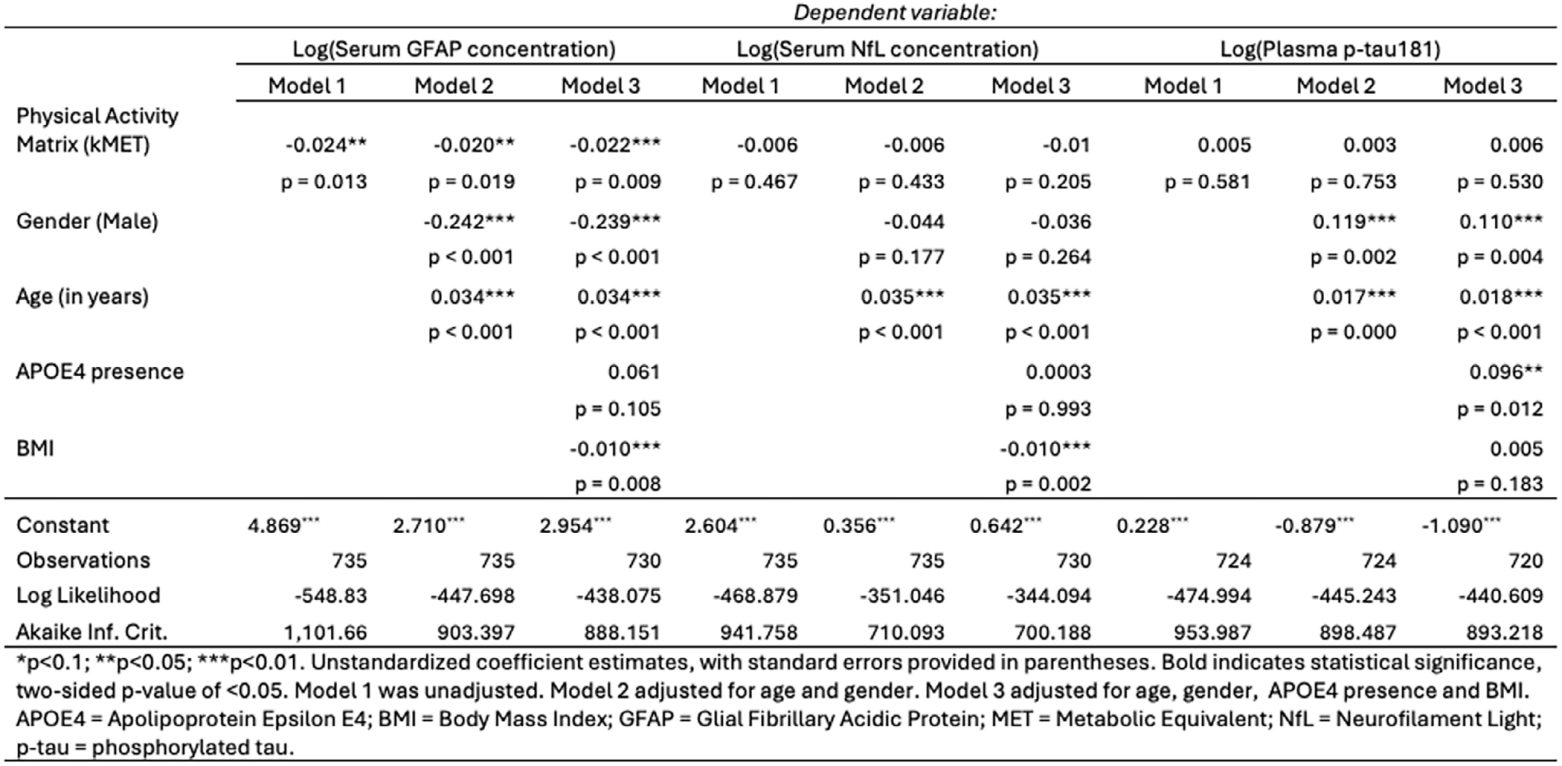# Physical activity and blood‐based biomarkers of neurodegeneration in community dwelling Australians from ISLAND (Island Study Linking Ageing and Neurodegenerative Disease)

**DOI:** 10.1002/alz70860_097355

**Published:** 2025-12-23

**Authors:** Eddy Roccati, Jessica M. Collins, Michele L Callisaya, Jane E. Alty, Anna E. King, James C. Vickers, James J.R. Brady

**Affiliations:** ^1^ Wicking Dementia Research and Education Centre, University of Tasmania, Hobart, TAS, Australia; ^2^ Menzies Institute for Medical Research, University of Tasmania, Hobart, TAS, Australia; ^3^ Royal Hobart Hospital, Hobart, TAS, Australia; ^4^ University of Tasmania, Hobart, TAS, Australia

## Abstract

**Background:**

With three Lancet Commissions finding an increasing proportion of dementia cases could be prevented through lifestyle modification, there are now 14 modifiable risk factors accounting for approximately 45% of all cases. Physical activity, a key mid‐life modifiable risk factor for dementia, has been associated with cerebral and cerebrospinal biomarkers of Alzheimer's disease (AD), but less is known about the association of physical activity with blood‐based biomarkers of AD. In this large‐scale community cohort study, we aimed to investigate whether physical activity was associated with the hallmark blood‐based biomarkers of AD, and whether presence of apolipoprotein E epsilon 4 (APOE‐ε4) moderated this relationship.

**Method:**

739 cognitively healthy participants from ISLAND (Island Study Linking Ageing and Neurodegenerative Disease) completed a battery of online surveys, including background health, medications, demographic and validated physical activity questionnaires. Physical activity was assessed based on daily metabolic equivalent (MET) of task, Examples of light, moderate and vigorous were walking, bicycling and running respectively. ISLAND participants provided an intravenous blood sample which was analysed for serum neurofilament light (NfL), serum glial fibrillary acidic protein (GFAP), plasma phosphorylated tau 181 (*p*‐tau 181) and genotyped for the APOE‐ε4 allele (Figure 1). Generalized linear regression models were deployed to investigate the association of the physical activity matrix score (MET) and biomarkers of AD (GFAP, NfL and *p*‐tau 181).

**Result:**

Greater physical activity was significantly associated with serum GFAP (pg/mL). Sub‐group analysis revealed this association was moderated by presence of APOE‐ε4: APOE‐ε4 negatives had a significant association between MET and serum GFAP, but (Figure 2). Post‐hoc testing revealed that participants self‐reported levels of vigorous activity had the strongest association with serum GFAP, whilst light and moderate activity were not significant. There was no relationship between physical activity and *p*‐tau 181 or NfL.

**Conclusion:**

This study provides novel evidence for the association of physical activity with blood‐based biomarkers of AD, and how APOE‐ ε4 presence modifies this relationship. Physical activity offers an ideal target for large‐scale targeted community interventions seeking to reduce the risk of dementia and AD, as well as the biological prodrome that precedes clinical manifestation.